# Identification of key genes affecting porcine fat deposition based on co-expression network analysis of weighted genes

**DOI:** 10.1186/s40104-021-00616-9

**Published:** 2021-08-20

**Authors:** Kai Xing, Huatao Liu, Fengxia Zhang, Yibing Liu, Yong Shi, Xiangdong Ding, Chuduan Wang

**Affiliations:** 1 Animal Science and Technology College, Beijing University of Agriculture, Beijing, China; 2 Key Laboratory of Animal Genetics, Breeding and Reproduction, Ministry of Agriculture, National Engineering Laboratory for Animal Breeding, College of Animal Science and Technology, China Agricultural University, Beijing, China

**Keywords:** Fat deposition, Pigs, RNA-seq, WGCNA

## Abstract

**Background:**

Fat deposition is an important economic consideration in pig production. The amount of fat deposition in pigs seriously affects production efficiency, quality, and reproductive performance, while also affecting consumers’ choice of pork. Weighted gene co-expression network analysis (WGCNA) is effective in pig genetic studies. Therefore, this study aimed to identify modules that co-express genes associated with fat deposition in pigs (Songliao black and Landrace breeds) with extreme levels of backfat (high and low) and to identify the core genes in each of these modules.

**Results:**

We used RNA sequences generated in different pig tissues to construct a gene expression matrix consisting of 12,862 genes from 36 samples. Eleven co-expression modules were identified using WGCNA and the number of genes in these modules ranged from 39 to 3,363. Four co-expression modules were significantly correlated with backfat thickness. A total of 16 genes (*RAD9A*, *IGF2R*, *SCAP*, *TCAP*, *SMYD1*, *PFKM*, *DGAT1*, *GPS2*, *IGF1*, *MAPK8*, *FABP*, *FABP5*, *LEPR*, *UCP3*, *APOF*, and *FASN*) were associated with fat deposition.

**Conclusions:**

*RAD9A*, *TCAP*, *SMYD1*, *PFKM*, *GPS2*, and *APOF* were the key genes in the four modules based on the degree of gene connectivity. Combining these results with those from differential gene analysis, *SMYD1* and *PFKM* were proposed as strong candidate genes for body size traits. This study explored the key genes that regulate porcine fat deposition and lays the foundation for further research into the molecular regulatory mechanisms underlying porcine fat deposition.

**Supplementary Information:**

The online version contains supplementary material available at 10.1186/s40104-021-00616-9.

## Background

Pork is an important and widely used animal resource and has become one of the main sources of protein in human diets. Fat deposition is economically important in pig production and is closely related to production efficiency [1], pork quality [2], and reproductive characteristics [3]. It also affects consumer choice. Fat deposition-related traits are quantitative traits with certain genetic variations. Identifying molecular markers that affect porcine fat deposition is an important way of accelerating genetic progress. Because they have similar genetic and physiological characteristics as humans, domestic pigs have become ideal animal models for studying obesity and metabolic syndrome in humans [4]. Therefore, the molecular mechanisms underlying porcine fat deposition have always been a focal point in scientific research.

Animal fat mainly exists in the form of subcutaneous fat, visceral fat, intramuscular fat, and intermuscular fat, and there is a strong positive correlation in the characteristics of fat deposition. Pig backfat thickness has a high genetic and phenotypic correlation with body fat rate, subcutaneous fat thickness, and intramuscular fat content, so it can be used as an indicator of how much fat is deposited in the body [4]. Adipose deposition is a dynamic equilibrium process that primarily includes the synthesis, decomposition, and transport of fat. These processes occur mainly in adipose tissue, liver, and muscle [5]. Therefore, in order to investigate the formation and development of fat in this study, pig backfat thickness was used as a phenotype for the selection of samples from these three tissue types.

The transcriptome represents the overall level of gene expression in a sample (cell, tissue, etc.) and is therefore referred to as the “expression profile.” With the development of second-generation sequencing technology, there have been many studies on the transcriptome of porcine fat-related traits using RNA-seq technology. Li et al. [6], Wang et al. [7], Sodhi et al. [8], and Xing et al. [9] used transcriptomic data for differential expression analysis and found a large number of genes associated with fat deposition and metabolism in pigs. Hundreds of genes have been shown to be associated with pig fat development. However, organisms are complex systems, and the genes involved in the regulation of life activities are interconnected to form a complex network system, so the relationship between thousands of genes in multiple tissues must be considered while studying the developmental characteristics and mechanisms of tissues. Differential expression analysis may fail to detect important biological pathways or gene-gene interactions associated with disease because it focuses on the effects of individual genes rather than on the effects of gene networks. Weighted gene co-expression network analysis (WGCNA) is an efficient and accurate method for bioinformatics and biodata mining [10]. Co-expressed genes often form densely connected subgraphs in the network (generally corresponding to gene groups or signal pathways with similar functions) and have specific biological functions, forming local substructure modules [11, 12]. The interaction between genes can be revealed at the system level, helping researchers to further understand the mechanisms behind gene interactions and find the regulatory center of co-expressed genes [13]. The discovery of modules that are highly correlated with target traits is important for the rapid identification of key genes associated with these traits [14]. In a variety of biological fields, including cancer studies, genetics, and brain imaging, WGCNA has been widely used and is useful for identifying candidate biomarkers or therapeutic targets [15]. In recent years, WGCNA has been increasingly used to study pigs, with several reports demonstrating its effectiveness [16].

This study aimed to use transcriptome data from multiple tissues to construct co-expression modules and identify modules that co-express genes associated with fat deposition. The modules of interest were analyzed in combination with differentially expressed genes and the core genes in each module were identified. This study provides a starting point for further exploration of the molecular regulatory mechanisms underlying fat deposition.

## Methods

### Experimental material

A total of 500 purebred Landrace and Songliao black sows from the same pig farm were selected for the study. All of them were healthy and reared in the same environment. The backfat thickness of each living pig was measured. It was measured 5 cm from the dorsal midline, between the third and fourth last ribs, using a B-ultrasound machine. The backfat thicknesses of pigs weighing 100 kg body were corrected using the backfat thickness correction formula. High backfat thickness and low backfat thickness populations were selected from each breed (Stable 1). After slaughter, subcutaneous fat, longus dorsal muscle, and liver samples were collected, and total RNA was extracted for transcriptome sequencing. Specific sample information is presented in Table 1. The following detailed sequencing steps were used: Oligo (dT) magnetic beads were used to adsorb and purify mRNA, and Oligo (dT) primer-guided reverse transcription was used to synthesize double-stranded cDNA. Then, using exonuclease and polymerase, the nucleotide ‘A’ was added to the 3′ end of the DNA fragments, specific paired-end (PE) adaptors was connected, and the cDNA fragments with the adaptors were separated and added for recovery. Amplification with a PCR primer cocktail (10 cycles) was used to concentrate cDNA, and PCR products were purified using the AMPure XP system (Beckman Coulter, Beverly, MA, USA). The quality of the transcriptomic sequence library was then tested using an Agilent Bioanalyzer 2100 (Agilent, USA). The TruSeq PE Cluster kit V3-C Bot-HS (Illumina) was used for password clustering on the C Bot Cluster Generation system, and the Illumina Hiseq 2000 sequencing platform was used for PE sequencing. The sequencing reads generated were 110 bp long.

**Table 1:** Sample information

ID	Breed	Group^a^	Tissue	ID	Breed	Group	Tissue
S_H_1_F	Songliao	High	Fat	L_H_1_F	Landrace	High	Fat
S_H_2_F	Songliao	High	Fat	L_H_2_F	Landrace	High	Fat
S_H_3_F	Songliao	High	Fat	L_H_3_F	Landrace	High	Fat
S_L_1_F	Songliao	Low	Fat	L_L_1_F	Landrace	Low	Fat
S_L_2_F	Songliao	Low	Fat	L_L_2_F	Landrace	Low	Fat
S_L_3_F	Songliao	Low	Fat	L_L_3_F	Landrace	Low	Fat
S_H_1_L	Songliao	High	Liver	L_L_1_L	Landrace	Low	Liver
S_H_3_L	Songliao	High	Liver	L_L_2_L	Landrace	Low	Liver
S_L_1_L	Songliao	Low	Liver	L_L_3_L	Landrace	Low	Liver
S_L_3_L	Songliao	Low	Liver	L_L_4_L	Landrace	Low	Liver
S_H_1_M	Songliao	High	Muscle	L_H_1_L	Landrace	High	Liver
S_H_3_M	Songliao	High	Muscle	L_H_2_L	Landrace	High	Liver
S_H_4_M	Songliao	High	Muscle	L_H_3_L	Landrace	High	Liver
S_L_1_M	Songliao	Low	Muscle	L_H_4_L	Landrace	High	Liver
S_L_3_M	Songliao	Low	Muscle	L_H_1_M	Landrace	High	Muscle
S_L_4_M	Songliao	Low	Muscle	L_H_2_M	Landrace	High	Muscle
L_L_1_M	Landrace	Low	Muscle	L_H_3_M	Landrace	High	Muscle
L_L_2_M	Landrace	Low	Muscle	L_L_3_M	Landrace	Low	Muscle

^a^Backfat thickness

The following pairs of individuals are full siblings: S_H_1/S_L_1, S_H_2/S_L_2, S_H_4/S_L_4, L_H_1/L_L_1, L_H_2/L_L_2, and L_H_3/L_L_3

To process transcriptome sequence data, IlluQC.pl (NGS QC Toolkit) [17] was used for quality control of the sequencing reads, and reads with unknown sequences greater than 10% and quality scores less than 20 were removed. HISAT2 [18] was used for fast and accurate sequence alignment. Finally, a transcriptome gene expression count file was converted using Samtools [19] and featureCounts [20] to obtain the gene expression profile in each tissue sample.

### Gene expression matrix construction

Gene expression data from 36 samples were combined to construct an expression count matrix. From a biological point of view, only after gene expression reaches a certain level can the related protein be translated to perform the biological function [21]; therefore, low-expression data (sum counts < 10) were eliminated from analysis. The gene expression matrix used for WGCNA was obtained by normalizing the variance stabilizing transformation (VST) [22] function in the DESeq2 package [23].

### Differential gene expression analysis

The gene expression count matrix was divided into groups based on backfat thickness (high and low). Differential expression analysis was conducted for different tissues from the two breeds based on gene expression count by following the outlined steps: a) differential gene expression analysis was conducted for different fatty tissues from Landrace pigs; the differential genes of high backfat thickness and the second backfat thickness were compared, and the same method was also used to analyze the liver tissues and muscle tissues of Landrace pigs, b) differential expression analysis was conducted for different tissues from Songliao black pigs, c) the differentially expressed genes from different tissues of the same variety were combined as candidate gene sets affecting fat deposition in this variety, d) the genes in the intersecting candidate gene sets from different breeds were selected as candidate genes affecting pig fat deposition (Supplementary Fig. 2 and Supplementary Fig. 3). Analysis of differential expression between different subgroups was conducted using DESeq2 v.1.20 [23]. DESeq2 performs internal normalization, where the geometric mean is calculated for each gene across all samples. The counts for each gene in each sample were then divided by this mean. The median of these ratios in a sample is the size factor for that sample. This procedure corrects for library size and RNA composition bias, which can arise, for example, when only a small number of genes are highly expressed under one experimental condition and not in another. Additionally, DESeq2 automatically detects count outliers using Cook’s distance and removes these genes from the analysis. DESeq2 uses shrinkage estimation for dispersions and fold changes. The dispersion value was estimated for each gene using a model fit procedure. Using these estimations, the package fits a negative binomial generalized linear model for each gene and uses the Wald test for significance testing. Genes with *P* values less than 0.05 were selected as differentially expressed genes. Differential gene expression analysis was conducted as recommended in the Bioconductor software (http://bioconductor.org/help/workflows/rnaseqGene/).

### Weighted co-expression network construction

The pig gene co-expression network was constructed using the WGCNA package in R [24]. Outlier samples are likely to have adverse effects on the results of network module analyses. Therefore, it was necessary to first identify and remove outlier samples before constructing the network. No outlier samples were found by clustering the samples, and all 36 samples were retained. The final expression matrix of 12,862 probes was used to construct the co-expression network. A one-step method was used to construct a network and determine the gene module. Based on the description by Zhang et al. [10], gene co-expression networks should have scale-free characteristics and follow a power-law distribution. A weighted adjacency matrix was created, defined as *A*_*ij*_ = *|cor* ^*^ *(x*_*i,*_ *x*_*j*_*) |*^*β*^, where *x*_*i*_ and *x*_*j*_ are the *ith* and *jth* genes, respectively. Adjacent to the adjacent network is the combination of the soft thresholding power parameter *β*, which is required to improve the co-expression similarity for computing the adjacency. To keep the network consistent with scale-free topology, the pickSoftThreshold() function was used to analyze the network topology and choose an appropriate soft-thresholding power value (*β*) to build the network and allow the mean connectivity of all genes in the module to be evaluated. The soft thresholding power parameter, *β*, was set to 14 following a sensitivity analysis of the scale-free topology. By selecting an appropriate soft threshold, the correlation coefficients in the similarity matrix can be continuously transformed into an exponential function transformation to obtain the adjacency function. The adjacency matrix was subsequently converted to a topological overlap matrix (TOM), which can evaluate the direct correlation of gene pairs and their degree of agreement with other genes in the dataset [25]. The division of gene modules was based on the degree of connection between modules, so it was necessary to convert the degree of coincidence of topological connections into the degree of divergence. Average linkage hierarchical clustering was conducted in accordance with the TOM-based dissimilarity measure. For modules with high topological overlap (dissimilarity less than 0.25), the adaptive dynamic pruning algorithm was used to merge the modules, and the gene modules were then recalculated. For the gene dendrogram, a minimum gene module size of 30 was used to classify similar genes into one module [15]. The module eigengene (ME), which can be regarded as representative of the gene expression profiles of a module, is defined as the first principal component of a module of interest. Associating gene modules with phenotypic information is helpful for finding gene modules related to target traits; genes in these modules are likely to be important for trait expression. Module eigengenes can summarize the expression patterns of all genes into a single characteristic expression profile within a given module. Therefore, the correlation between phenotypic characteristics and each ME was one of the factors used to determine the key module. This study establishes the relationship between the sample information and the module by constructing a matrix; if the rows and columns were the same samples, the value of the matrix was 1; if not, it was 0. Finally, the correlation coefficient between the matrix and ME module was calculated.

We also calculated the gene significance (GS) and module significance (MS). The equation *GSi* = |*cor (xi, T)*| was used to quantify the gene *i* of GS, where *xi* is the expression profile of *i*, and *T* is a sample trait [24]. In key modules, MS was identified as another factor, defined as the average significance of all genes contained in a module. Generally, the higher the MS value, the higher the correlation between the module and the sample type. Module membership (MM) is the correlation coefficient between this gene and the trait characteristic genes of this module and can be used to screen important genes in the module. Genes with high module membership often also have high gene significance in modules related to a trait of interest. Genes with high module membership in modules related to traits are natural candidates for further validation. If GS and MM show a very significant correlation for a gene in a module, it means that the central genes in that module also tend to be highly correlated with the target trait. This would not only indicate the accuracy of the classification, but would also allow important genes to be identified in this way.

The highly connected genes in the module are also known as hub genes, which may play an important role in the module [26]. Hub genes are conserved to a certain extent and are at the core of the gene co-expression network and can act as a genetic buffer to reduce the impact of other gene mutations [27]. We identified the top 30 hub genes in the module that were most closely related to backfat thickness differences, that is, the 30 genes with the highest connectivity in the module, and used Cytoscape software [28] to map the gene-gene interaction network for the visualization of gene relationships.

### Functional enrichment analysis of co-expression modules and selection of candidate genes

Gene Ontology (GO; http://www.geneontology.org/) is widely used in the field of bioinformatics and classifies genes into terms from three different biological categories: cellular components (CC), molecular functions (MF), and biological processes (BP). The default parameters were used to identify the phenotype-related module, and three gene ontology enrichment analyses were performed on the genes in the module. False discovery rate (FDR) *P*-values less than 0.05 were considered significant, and the 10 most prominent entries for each analysis were kept. The Kyoto Encyclopedia of Genes and Genomes (KEGG, http://www.genome.jp/kegg/) is a database for the systematic analysis of gene function and genomic information, which helps researchers study genes and gene expression as part of a whole network. “ClusterProfiler” [29] and the “ggplot2” packages were used to analyze and visualize the genetic information, respectively. The R software package BioMart (http://www.biomarbiomart.org/) [30] was used to annotate genes in the module, using the reference genome Sscrofa11.1. We selected a subset of modules based on their functional annotation and selected genes related to fat development. Based on the above information, the candidate genes affecting fat growth and development in this experiment were identified.

For differentially expressed genes, we conducted enrichment analysis to identify differences between different tissues. The overall analysis, including GO and KEGG, was carried out in KOBAS 3.0 (http://kobas.cbi.pku.edu.cn/kobas3/?t=1).

### Quantitative real-time PCR (qPCR)

To confirm the sequence data, reverse transcription quantitative real-time PCR was performed using the Light Cycler® 480 Real-Time PCR System (Roche, USA). Nine genes (three DEGs in each tissue) were selected to validate the changes in mRNA expression between different groups. Total RNA from samples that were used for high-throughput RNA-seq was isolated and converted into cDNA using the Revert Aid™ First Strand cDNA Synthesis Kit (Thermo Fisher Scientific Inc., USA). A reaction volume of 20 μL was used in the qPCR reactions according to the manufacturer’s protocol. qPCR experiments were performed in triplicate, and the average Ct was used for further analysis. The 2^−ΔΔCt^ method was used to determine relative mRNA abundance.

## Results

### Construction of co-expression modules

**Fig. 1:**
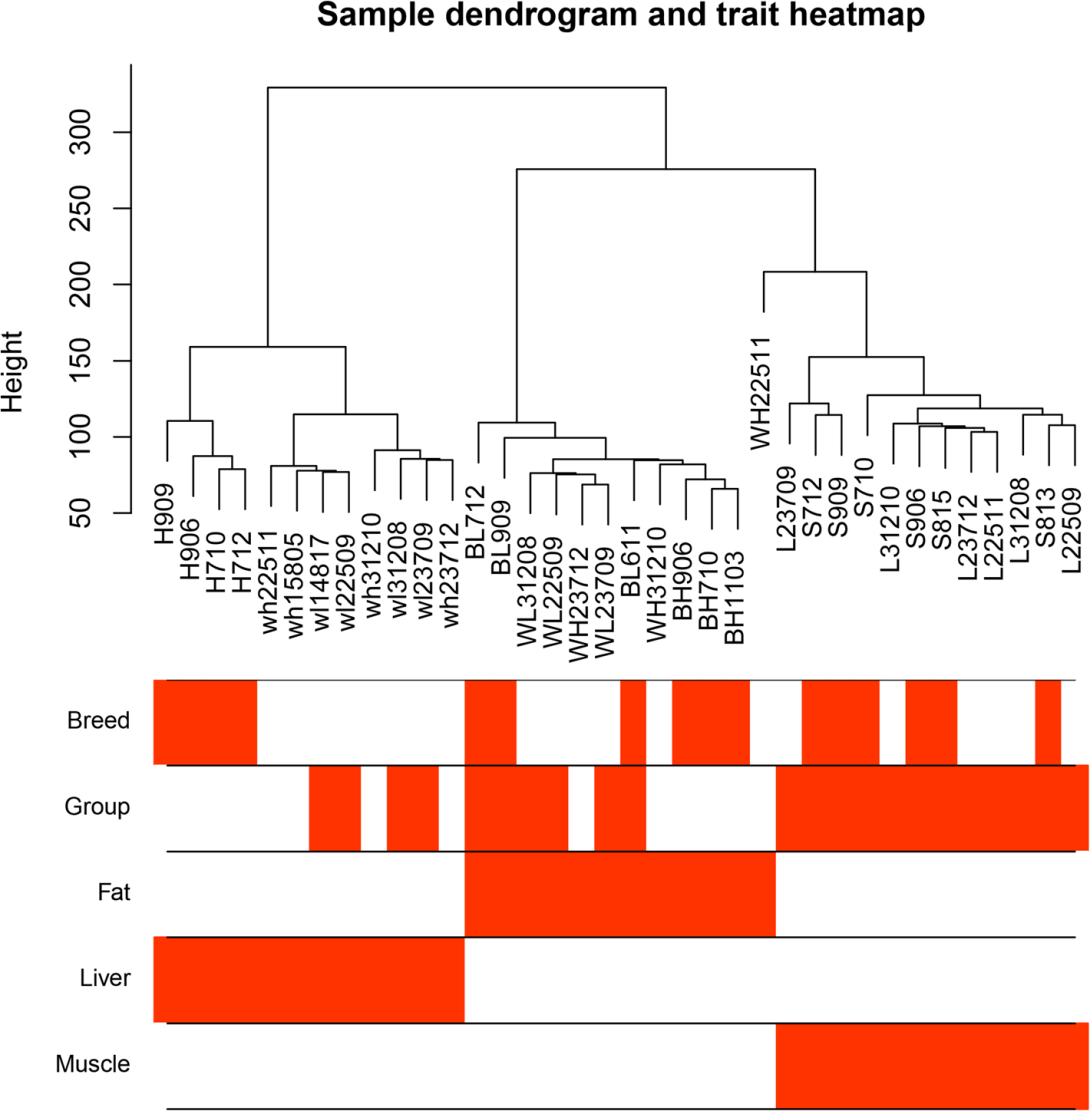
Sample clustering. Clustering was conducted to eliminate low-quality samples. There were no outliers in the clustering tree

We used RNA-seq of different tissues from Songliao black and Landrace pigs to construct the gene expression matrix consisting of 12,862 genes from 36 samples after standardized processing. The WGCNA package tool was used to construct co-expression modules. No outlier samples were found in the hierarchical clustering of samples when they were analyzed using the flashClust tools package (Fig. 1).

**Fig. 2:**
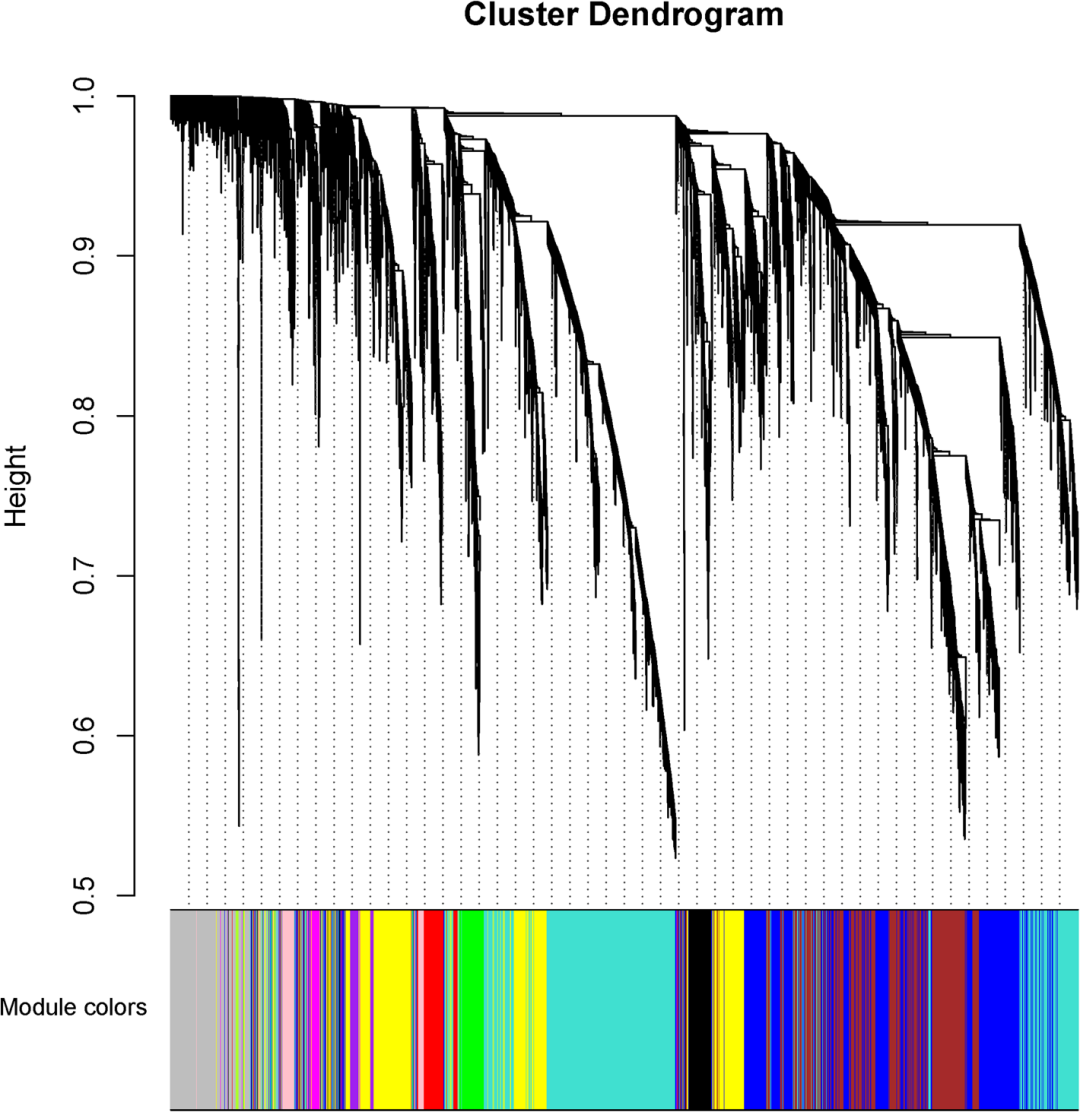
Division of gene modules. The figure shows the clustering of genes, and the division of gene modules is based on this result. Branches of the same color were divided into the same gene modules

Based on the standard of a scale-free network, we choose the appropriate weighted parameter of the adjacency function, namely, the soft threshold. This selection can make the adjacency function satisfy the scale-free condition, which primarily affects the independence and average connectedness of the common expression module. First, an appropriate soft thresholding power parameter was screened (Supplementary Fig. 2). When the power value was 14, the independence was approximately 0.8, and the average connectedness was relatively high. Then, we calculated the correlation matrix and adjacency matrix, combined into the topology matrix, and finally identified a total of 11 gene modules (Fig. 2; Table 2) based on genetic similarity (based on merging modules with dissimilarities less than 0.25 and a minimum module size of 30).

**Table 2:** The number of genes in each of the 11 modules

Module color	Frequency (number) of genes
Black	358
Blue	2,570
Brown	1,977
Green	416
Green-yellow	39
Grey (unassigned genes)	1,193
Magenta	174
Pink	277
Purple	172
Red	412
Turquoise	3,363
Yellow	1,910

### Analysis of the relationship between gene modules and phenotypic sample information

**Fig. 3:**
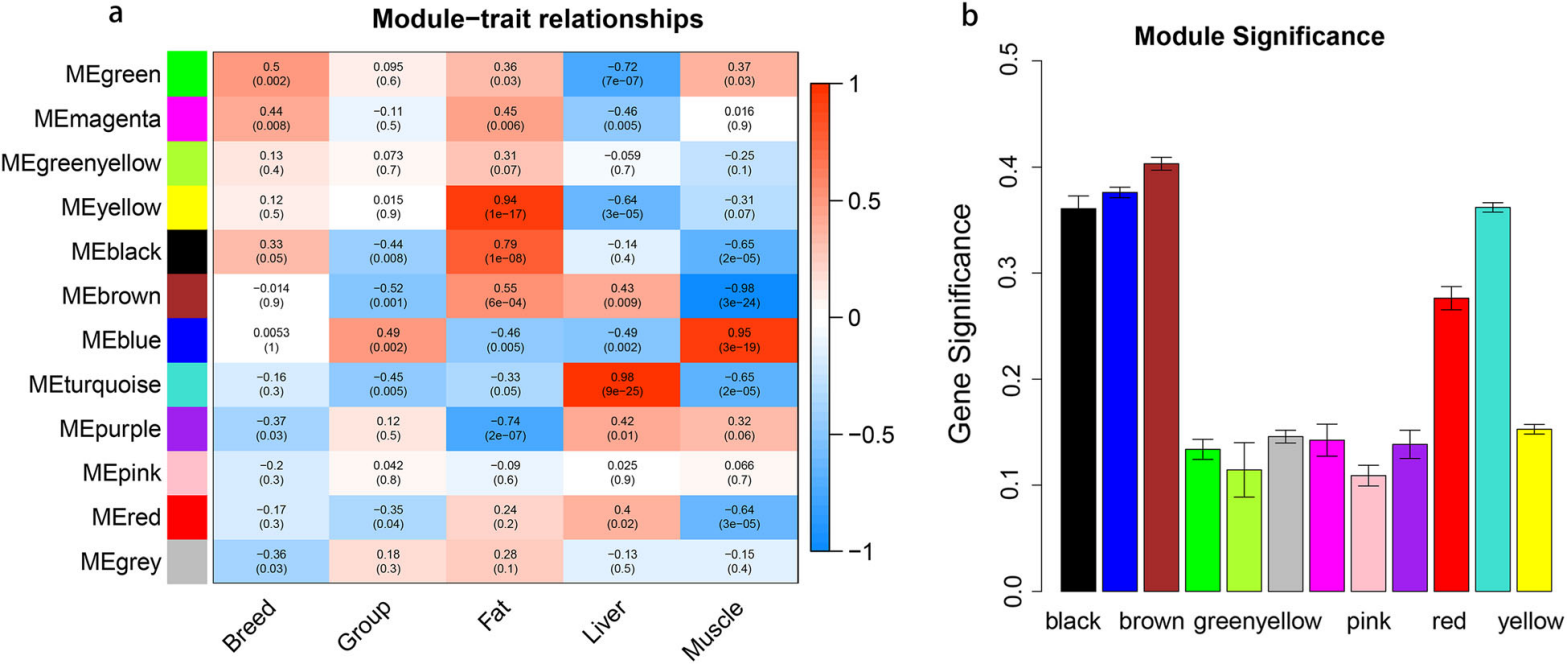
Module-trait associations. Panel **a** shows correlation between gene module and sample information, where the x-axis represents sample information, including varieties, grouping of backfat thickness, and sample tissue type. The y-axis represents each gene module. In the panel, the darker the color, the higher the correlation, with red representing positive correlation and blue representing negative correlation. The significance value, represented by the *P* value, is in brackets. Panel **b** shows the absolute correlation between the genes in each module and backfat thickness

Phenotypic information included sample breed, high or low backfat thickness, and organ type. The Pearson correlation coefficient between the eigengenes of modules and corresponding variables represents the correlation between the module and phenotypic information (Fig. 3). As shown in Fig. 3, the black, brown, and turquoise modules were moderately negatively correlated with backfat thickness (*r* = − 0.44, *r* = − 0.52, and *r* = − 0.45, respectively; *P* < 0.01), and moderately positively correlated with the blue module (*r* = 0.49; *P* < 0.01). All four modules were strongly correlated with different tissue types. It is postulated that the genes in these modules are involved in fat formation and have a function in different tissues. In addition, the yellow module showed a high positive correlation with the adipose tissue (*r* = 0.94). Adipose tissue is the organ most directly associated with fat formation, and its related genes deserve attention.

**Fig. 4:**
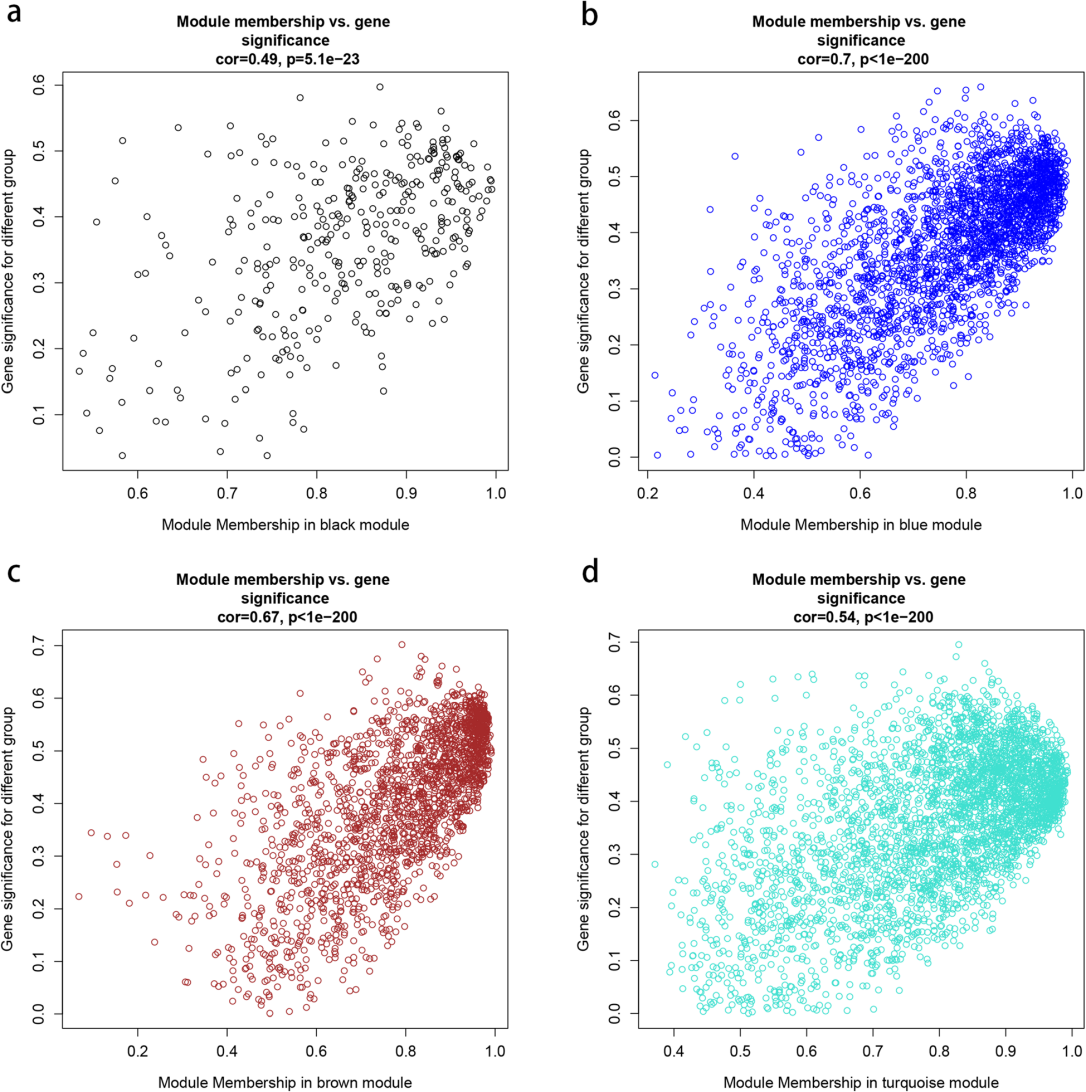
Scatterplot of Gene Significance (GS) for backfat vs. Module Membership (MM) in candidate modules. The figure shows the significance of the genes in the four modules. The x-axis represents the value of membership in each module, and the y-axis represents gene significance for backfat; **a**, **b**, **c**, and **d** represent the genes in the black, brown, blue, and turquoise modules, respectively. The gene in the upper right corner of each graph is the hub gene that we need to look for. These genes are highly correlated with phenotypes and have a high MM, which is a good representation of the gene module

A module eigengene (ME) is the first principal component gene in a specific module and represents the overall level of gene expression within the module. Module membership (MM) for a gene is the correlation between the expression profile of a sample and the ME expression profile of a certain feature’s associated gene. By creating a cluster dendrogram of genes and a heat map of the topological overlap between genes, the blue module was found to be most closely associated with fat formation. Therefore, we focused on the genes in this module for subsequent analyses. As a final module assessment, we drew scatter plots of GS for backfat (high or low) versus MM for the black, brown, blue, and turquoise modules (Fig. 4). In these modules, there is a significant correlation between GS and MM, indicating that genes tend to be highly correlated with backfat in modules associated with a trait of interest.

### Functional enrichment analysis of genes in relevant modules

**Fig. 5:**
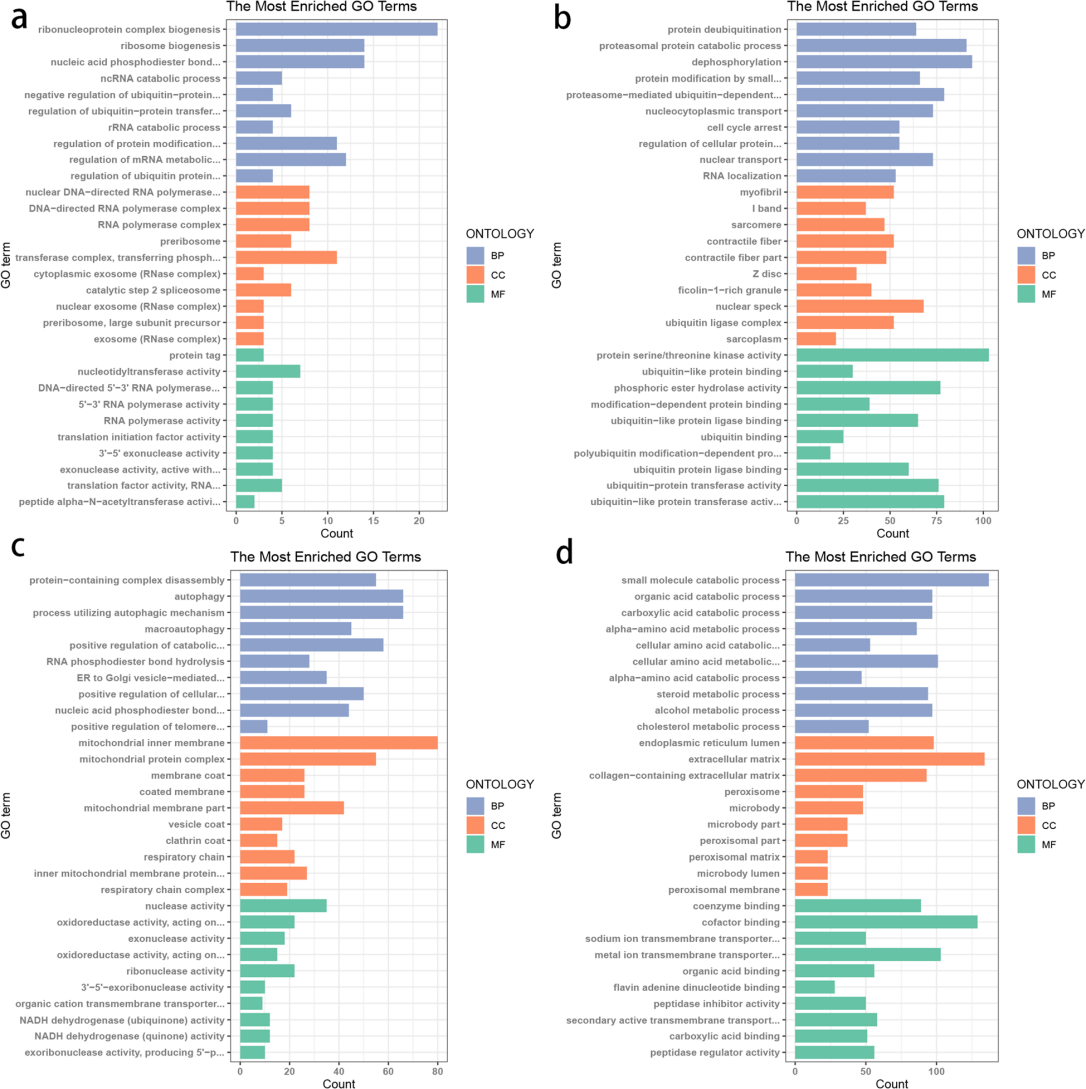
Gene ontology enrichment analysis for genes in co-expression modules. In the figure, **a**, **b**, **c**, and **d** show the enrichment of genes in black, blue, brown, and turquoise modules from three different biological categories, respectively. The y-axis represents the gene-enriched entries, and the x-axis represents the number of genes enriched in the same entry. In each panel, different biological categories are represented with different colors. CC, cellular components; MF, molecular functions; BP, biological processes

GO enrichment analysis was performed for genes in the black, brown, blue, and turquoise modules (Fig. 5). The 10 most significant terms in the BP, MF, and CC categories are shown in the figure, and the terms enriched by each of the four modules are different. The genes in the black module were concentrated in ribonucleoprotein complex biogenesis (GO:0022613), nuclear DNA-directed RNA polymerase complex (GO:0055029), and protein tag (GO:0031386). Genes in the blue module were associated with protein metabolism and muscle fiber formation, mainly located in protein deubiquitination (GO:0016579), myofibril (GO:0030016), and protein serine/threonine kinase activity (GO:0004674). The genes in the brown module were similar to those in the blue module, and were enriched in protein-containing complex disassembly (GO:0032984), inner membrane (GO:0005743), and nuclease activity (GO:0004518). The turquoise module had the highest number of genes, up to 3,363, which are involved in the processing of ribose and the transport of small molecules. Entries were mainly in the terms small molecule catabolic process (GO:0044282), endoplasmic reticulum (GO:0005788), and coenzyme binding (GO:0050662). Although these entries vary, the biological processes involved are associated with lipid formation. From KEGG analysis of the genes in these modules, we showed that the pathways with significant enrichment involved gluconeogenesis, insulin signaling pathway, MAPK signaling pathway, REDOX and pentose metabolism, and other important pathways (Supplementary Table 1). In addition, with regard to specific gene functions, we found differing numbers of genes related to the fat deposition process in these four modules and the yellow module (Table 3).

**Table 3:** Candidate genes from co-expression modules

Module Color	Candidate genes
Black	*RAD9A*
Blue	*IGF2R, SCAP, TCAP, SMYD1, PFKM*
Brown	*DGAT1, GPS2, IGF1, MAPK8*
Turquoise	*FABP4, FABP5, LEPR, UCP3, APOF*
Yellow	*FASN*

### Module visualization and hub genes

**Fig. 6:**
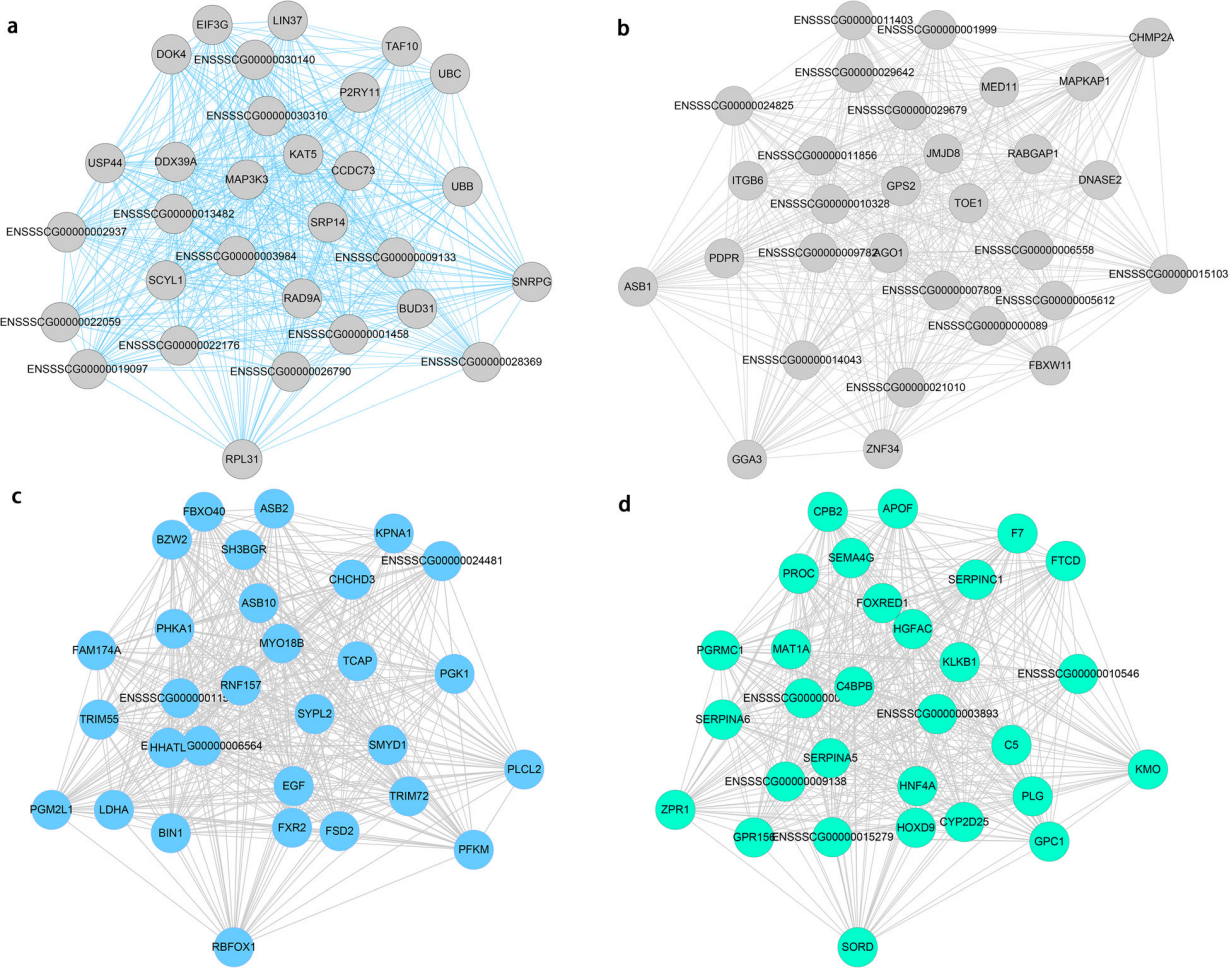
Visualization of the top 30 genes in specific modules: (**a**) black, (**b**) brown, (**c**) blue, and (**d**) turquoise. Network relationship diagram of hub genes in different modules. The thicker the line between genes, the higher the correlation of genes; the higher the number of lines between the gene and other genes, the more important the gene

Hub genes are a series of genes with the highest degree of connectivity in a module and that, to a certain extent, determine the characteristics of the module. Compared with hub genes in the global network, hub genes in the module tend to be more biologically significant. Intra-module analysis was performed to calculate the degree of connectivity between the genes. According to the instructions in the WGCNA package [24], intramodular connectivity measures the connection or co-expression of a given gene with respect to the genes of a particular module. Intramodular connectivity may be interpreted as a measure of module membership. For each module, the average degree of connectivity between genes was calculated; that is, the correlation between genes was measured. Finally, the average degree of connectivity between genes was calculated based on the absolute value of the correlation. The genes within a module were sequenced based on connectivity, and the 30 genes with the highest connectivity within each module were selected as hub genes. The top 30 genes with the highest degree of connectivity were selected from the four candidate modules, and Cytoscape software was used to generate the gene interaction network diagrams (Fig. 6). Based on the candidate genes associated with fat deposition (Table 3), we identified that in the black, blue, brown, and turquoise modules, the core genes that may be involved in the control of the fat deposition process are *RAD9*, *TCAP*, *SMYD1*, *PFKM*, *GPS2*, and *APOF*.

### Differentially expressed genes (DEGs)

The genes were further screened to identify DEGs, ensuring that the candidate genes were as differentiated as possible with regard to grouping based on backfat thickness.

Differential analysis was conducted for genes in the selected tissues from both pig breeds. In the Landrace pigs, there were 1,485 differentially expressed genes in the adipose tissue, 183 differentially expressed genes in the liver tissue, 1,888 differentially expressed genes in the muscle tissue, and a combined total of 2,334 differentially expressed genes. In the Songliao black pigs, there were 2,239 differential genes in the adipose tissue, 901 differential genes in the liver tissue, 2,459 differential genes in the muscle tissue, and a combined total of 3,626 differential genes. There were 1,123 common differentially expressed genes in the two breeds and 659 common genes in the four modules (Supplementary Fig. 3).

Differential gene enrichment analysis in different tissues showed that enrichment entries differed in the different tissues (Supplementary Fig. 4), with most of them involved in energy metabolism, including phosphate dephosphorylation, AMPK signaling pathway, and multiple entries with ATP keywords. Some of these terms, such as fatty acid degradation, fatty acid metabolism, and fat absorption, have been directly associated with fat deposition.

**Fig. 7:**
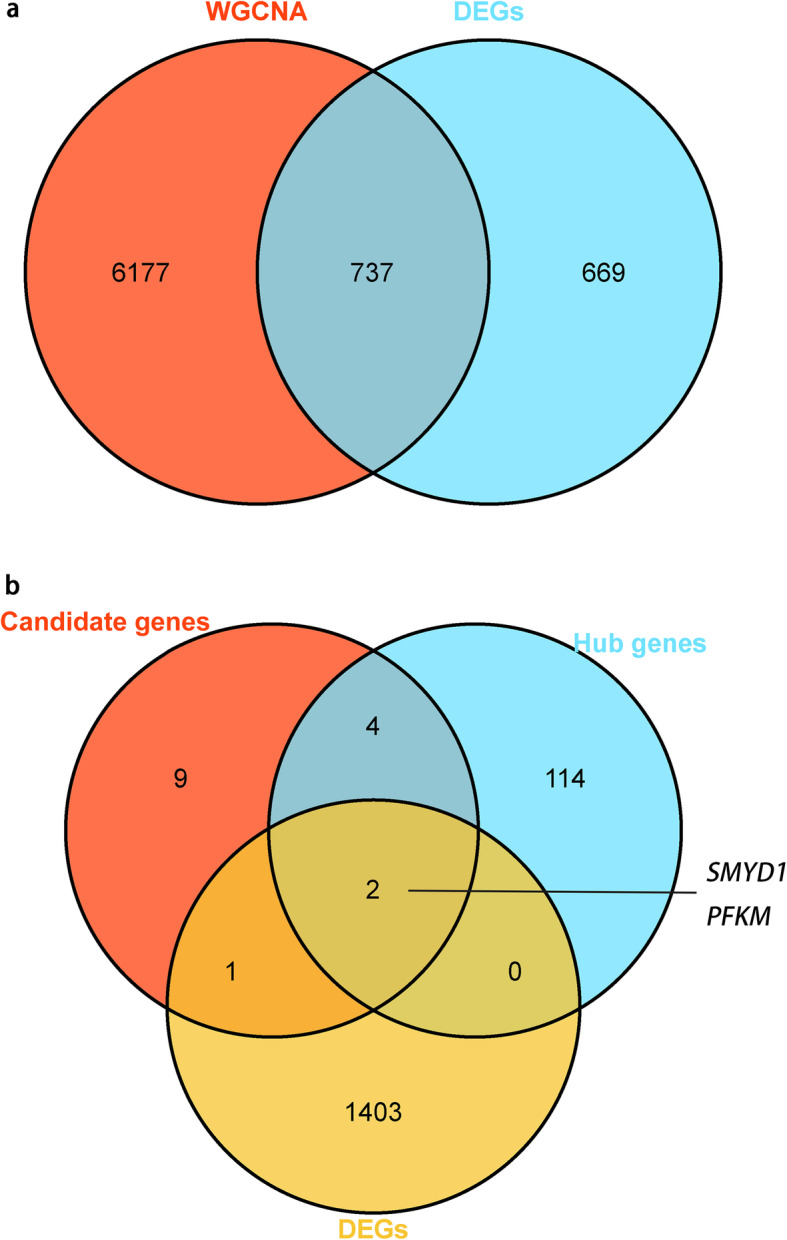
Volcanogram (**a**) and Venn map (**b**) of differentially expressed genes (DEGs). Panel **a** shows differential gene expression. The x-axis represents the multiple of differences, denoted by log2 Fold Change; the larger the absolute value, the larger the multiple of difference. The y-axis represents the significance of the difference, denoted by -log10 (*P*-value); the larger the value, the more significant the difference. The panel shows the names of the 20 genes with the most significant differences. Panel **b** shows the Venn map from various analysis methods, among which the intersection is the most important candidate gene

Among the 16 candidate genes identified via WGCNA, three genes, namely, *IGF2R*, *SMYD1*, and *PFKM,* were differentially expressed. *FASN* was also differentially expressed in the yellow module. In addition, two of the six hub genes, *SMYD1* and *PFKM*, were differentially expressed (Fig. 7).

### Quantitative real-time PCR (qPCR)

**Fig. 8:**
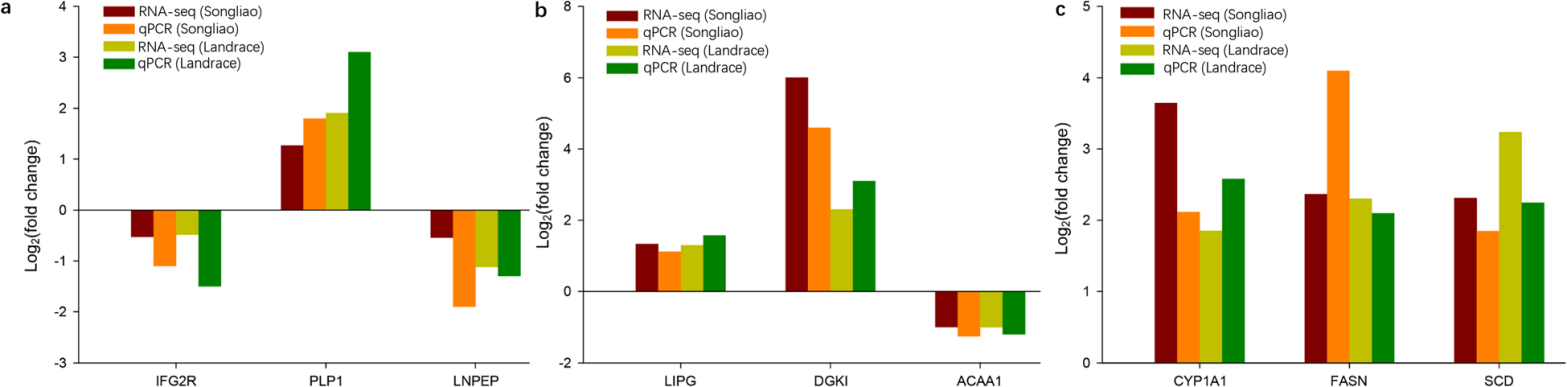
Validation of gene expression patterns obtained from RNA-seq data. Differentially expressed genes were validated in fat tissue (**a**), liver tissue (**b**), and muscle tissue (**c**) using qPCR

Nine genes were selected for RNA-seq data validation. Furthermore, the fold change in the selected genes showed similar trends. These results indicate that the DEGs identified using transcriptome data were efficient and reliable (Fig. 8).

## Discussion

Landrace is a widely used lean pig breed, while Songliao black pig is a fatty, Northeast China breed. These two breeds can well represent the whole pig breed in the study of fat deposition. In addition, similar regulation patterns were observed in different pig breeds. Therefore, these two breeds were selected to explore and study pig fat deposition, with the aim of identifying the key genes regulating pig fat deposition and providing a basis for improving pig production and meat quality through genetic intervention. Porcine fat deposition is a typical quantitative trait regulated by multiple genes, all of which interact with each other. In the traditional single-dimensional study of this trait, it is difficult to locate the main gene and its mechanism of action. However, WGCNA can make full use of phenotypic information to transform the associations between thousands of genes and phenotypes into associations between multiple gene sets and phenotypes, which can not only effectively reflect the interactions between genes, but also do not require multiple hypothesis testing and corrections [24, 31, 32]. Therefore, WCGNA was used to study the co-expression network in multiple groups (grouped by backfat thickness) in order to identify the major genes affecting fat deposition. The algorithmic principle of WGCNA network construction is to strengthen strong correlations and weaken weak correlations, so that the correlation value is more in line with the characteristics of a scale-free network and has more biological significance. The soft threshold is determined in order to make the network built more in line with the characteristics of the scale-free network, and the higher the R2, the better. The network constructed in this study met expectations, with R2 as high as 0.8, making the network of high biological significance. Meanwhile, the average connectivity was also high, reaching 185, indicating a high correlation between genes in the module and thus laying a foundation for locating key genes.

Functional enrichment analysis showed significant differences in the interactions between different modules. These were largely associated with their different functions, but were all involved in fat deposition and metabolism. The genes in the black module were primarily involved in the synthesis and processing of ribose and hydrolysis of phosphate bonds, which are closely associated with energy metabolism. An important source of fatty acids is the de novo synthesis pathway, which requires a large amount of energy. Glucose is the main precursor for fatty acid synthesis in monogastric animals, where glucose is decomposed and acetyl CoA is produced. Under the catalysis of acetyl CoA carboxylase (ACC) and fatty acid synthase (FAS), through a series of intermediate reactions, CoA enters the fatty acid synthesis system in the cytosol for a series of hydrogenation reduction processes, culminating in the synthesis of fatty acids. In pigs, most of this occurs in the fat tissue [33]. The black module was significantly associated with adipose tissue (0.79, *P* < 0.01), indicating that module division was rational and had biological significance. In the GO terms nucleic acid phosphodiester bond hydrolysis (GO:0090305), 3 ‘5’ exonuclease activity (GO:0008408), and exonuclease activity, active with either ribo-or deoxyribonucleic acid and phosphoesters (GO:0016796), the common gene *RAD9A* was found. This is not only related to the synthesis and hydrolysis of ATP, but also contributes to the control of cell differentiation, which affects the development of embryos and contributes to fatty cell differentiation [34].

The function of the genes in the brown module was similar to that of the genes in the black module, involved energy metabolism, and contained many terms associated with protein synthesis. This module was also significantly associated with fat and muscle tissue, both of which are major sites for energy synthesis and metabolism. The gene *IGF1* was found among the two significantly enriched terms: positive regulation of catabolic process (GO:0009896) and positive regulation of cellular catabolic process (GO:0031331). This gene encodes insulin-like growth factor-1, which has been found to be associated with fat formation in pigs [35]. Autophagy (GO:0006914), process utilizing autophagic mechanism (GO:0061919), macroautophagy (GO:0016236), and multiple signal-enrichment pathways (ssc04141: protein processing in endoplasmic reticulum), the common gene *MAPK8* (mitogen-activated protein kinase 8), is significantly correlated with backfat thickness in carcasses [36]. Acyl CoA: diacylglycerol acyltransferase (DGAT1) is a very important enzyme in mammals. It forms triacylglycerol by adding diacylglycerol to fatty acid acyl [37]. This enzyme plays an important role in lactation, small intestinal fat absorption, lipoprotein assembly, and fat formation [38, 39]. Noneman et al. [40] obtained the complete sequence of *DGAT1* in pigs and located *DGAT1* to chromosome 4 of pigs, mainly to study the correlation between this gene and obesity in pigs. The decrease in *GPS2* expression in adipocytes is closely associated with obesity [41, 42]. A previous study suggested that, in mice and humans, *GPS2* controls the reprogramming of white adipocytes, influencing pancreatic islet function and insulin secretion, thus affecting fat deposition [43]. In the blue module, *TCAP* exists in three significantly enriched terms: myofibril (GO:0030016), I band (GO:0031674), and sarcomere (GO:0030017). The protein TCAP (titin-cap; telethonin) is a myofilament protein that plays an important role in the assembly of myofibril, and is crucial for muscle growth and fat deposition [44]. The blue module was also highly correlated with muscle tissue (0.95, *P* < 0.01). Insulin-like growth factor (IGF) regulates lipid metabolism and affects the development of adipose tissue, mainly by binding to two types of insulin-like growth factor receptors (IGFR) on the cell surface [45]. In animal growth, IGFR is an important factor, particularly type I insulin-like growth factor receptor (IGF1R) and insulin-like growth factor receptor II (IGF2R) [46]. Several studies have shown that *IGF2R* affects fat deposition in pigs [47]. Additionally, SCAP/SREBP signaling is the main pathway regulating lipid metabolism [48]. Elevated blood glucose levels promote lipogenesis by activating SREBP transcription factors that are regulated by *SCAP* [49]. The *Smyd1* gene encodes a lysine methyltransferase that is specifically expressed in striated muscles. Muscle fiber atrophy has been observed in mice with mutations in this gene [50]. However, the development of intramuscular fat is closely associated with the state of muscle fibers, therefore, this gene is likely to be involved in the regulation of fat deposition. Phosphofructokinase, muscle type (PFKM), is a key regulatory enzyme that catalyzes the irreversible conversion of fructose-6-phosphate to fructose-1,6-bisphosphate during glycolysis. The porcine *PFKM* gene is expressed in skeletal muscles and heart, and its expression level is highly correlated with pork marbling and intramuscular fat content [51], which indicates that the expression of *PFKM* has a certain influence on the fat content of pigs.

The turquoise module is significantly associated with adipose and liver tissues, and there are five genes associated with fat deposition. *FABP4/FABP5* genes (fatty acid binding proteins 4 and 5) are directly involved in the regulation of fat deposition, are mainly expressed in fat and bone marrow cells, and are related to the development of insulin resistance [52, 53]. Third, many studies have shown that the polymorphism in *LEPR* is associated with the levels of blood glucose, insulin, leptin, and triglyceride, and *LEPR* deficiency can directly lead to the accumulation of fat, resulting in obesity [54, 55]. Fourth, the gene *UCP3* codes for a proton transport carrier distributed in the inner mitochondrial membrane that can reduce the H^+^ electrochemical gradient on both sides of the membrane, resulting in uncoupling of the oxidation and ADP phosphorylation processes. This is an important way for the body to produce energy and heat [56]. In pigs, the *UCP3* gene’s 5’ and 3’ sequence control regions, AvaI enzyme loci were significantly associated with fat metabolism [57]. Finally, experiments on mice have found that the *APOF* gene controls the speed of LDL (Low Density Lipoprotein) clearance mechanisms, thus affecting fat deposition [58].

In addition, although the yellow module was not associated with the candidate module grouping, it was highly correlated with the adipose tissue. This indicates that, although genes in this module may not be significantly associated with backfat thickness, they play a crucial role in the development and formation of fat, and the specific regulatory mechanism underlying this needs to be studied further. The candidate gene identified in the yellow module was *FASN*, which encodes fatty acid synthase (FASN), an enzyme involved in fat deposition and fatty acid composition [59, 60]. The genes in each module were analyzed. The importance of a gene is represented by the sum of its connectedness to all other genes in the module. The higher the total connectivity, the higher the correlation between this gene and all other genes, therefore, the greater the influence of this gene on other genes in the module and any change to this gene will change the module as a whole. In combination with the above analysis, hub genes (*RAD9A*, *TCAP*, *SMYD1*, *PFKM*, *GPS2*, and *APOF*) that are significantly associated with fat deposition were identified in four modules.

We conducted differential expression analysis on three tissues in the two pig breeds, and the results showed that the enrichment in different tissues were mostly different, indicating that the three tissues had functional differences with obvious tissue specificity (Supplementary Fig. 4). However, these differences are associated with energy metabolism and many similar terms are associated with the formation and metabolism of fatty acids. Therefore, it can be inferred that all three tissues are involved in fat deposition but have different divisions of labor, which is consistent with the complex process of fat deposition. Based on the similarity described by enrichment terms, we found that although there were differences in the same tissue function between Landrace and Songliao Black pigs, the specificity between tissues was significantly greater than that between breeds. This further indicates that there is little difference in fat deposition between these two breeds, and also reflects the rationality of the analyses used in this experiment. Based on the overlap between the common differentially expressed genes in the two breeds and the results of WGCNA (Fig. 7), more than half of the DEGs were identified by the WGCNA analysis, inferring similarity between the two analysis methods and further demonstrating the reliability of the results of this study. However, some of the fat-associated genes identified through WGCNA were not differentially expressed, suggesting that WGCNA could identify more information by establishing associations between genes, which is consistent with the description of WGCNA. To effectively reduce the number of candidate genes, we chose the overlap of hub genes found by WGCNA and differentially expressed genes as strong candidate genes, while candidate genes of other non-differentially expressed genes can also be used as references. Of the 16 candidate genes screened using WGCNA, six (*RAD9A*, *TCAP*, *SMYD1*, *PFKM*, *GPS2*, and *APOF*) were hub genes corresponding to each module associated with this trait, which play a major role in the regulation of fat deposition. In addition, we found that *SMYD1* and *PFKM* are also DEGs, further supporting the importance of *SMYD1* and *PFKM* in fat deposition.

## Conclusions

Four important modules were screened using WGCNA and 16 of these (*RAD9A*, *IGF2R*, *SCAP*, *TCAP*, *SMYD1*, *PFKM*, *DGAT1*, *GPS2*, *IGF1*, *MAPK8*, *FABP*, *FABP5*, *LEPR*, *UCP3*, *APOF*, and *FASN*) were associated with fat deposition. *SMYD1* and *PFKM* were not only hub genes but also differentially expressed genes, and are as strong candidate genes affecting fat deposition.

## Supplementary Information

**Additional file 1: Supplementary Table 1.** Body weight and backfat thickness between different groups. **Supplementary Table 2.** KEGG analysis of genes in the four modules related to traits. **Supplementary Fig. 1.** Analysis of network topology for various soft-thresholding powers. According to the definition of soft threshold, try to choose a large R^2^ value. According to the suggestion of WGCNA package, choose an R^2^ value greater than 0.8, that is, the value above the red line in the left figure. The figure on the right shows the average connectivity of the constructed network. The larger the network is, the closer the gene is, and the more conducive it is to screen out hub genes. **Supplementary Fig. 2.** Venn map of differentially expressed genes (DEGs) of Panel **a**, **b** and **c** respectively shows the situation of the differentially expressed genes in adipose tissue, muscle and liver of Songliao black pigs. Panel **e**, **f** and **g** respectively shows the situation of the differentially expressed genes in adipose tissue, muscle and liver of Landrace. The x-axis represents the multiple of difference, which is denoted by log2FoldChange. The larger the absolute value is, the larger the multiple of difference is. The y-axis represents the significance of the difference, which is denoted by -log10(*P*-value). The larger the value is, the more significant the difference is. Each panel shows the names of the top 20 genes with the most significant differences. **Supplementary Fig. 3.** Volcanogram of differentially expressed genes in different tissues of two breeds. Panel **a** and **b** respectively shows the overlap of the differentially expressed genes in adipose tissue, muscle and liver of Songliao black pigs and Landrace. Panel **b** shows the overlap of all the differentially expressed genes of Songliao black pigs and Landrace. **Supplementary Fig. 4.** GO enrichment analysis and KEGG pathway analysis in different tissues of two breeds. Panel **a, b** and **c** respectively shows the enrichment entries of the differentially expressed genes in adipose tissue, liver and muscle of Landrace. Panel **d, e** and **f** shows the enrichment entries of the differentially expressed genes in adipose tissue, liver and muscle of Songliao black pigs. The x-axis represents the significance of the difference, which is denoted by -log2(P-value). Each bubble represents an enriched function, and the size of the bubble is set with six gradients according to the *p*-value, from small to large, representing the different significance levels: ns (*P*-value> = 0.05), * (0.01 < =*P*-value< 0.05), ** (0.001 < =*P*-value< 0.01), *** (0.0001 < =*P*-value< 0.001), **** (1e-10 < =*P*-value< 0.0001), ***** (*P*-value<1e-10). The color of the bar is the same as the color in the circular network, which represents different clusters. For each cluster, if there are more than 5 terms, top 5 with the highest enrich ratio will be displayed.

## Data Availability

The datasets used during the current study are available from the corresponding authors on reasonable request. The RNA sequencing data for this study can be found in the NCBI Sequence Read Archive (SRA) under Bioproject: PRJNA407236, PRJNA234465, PRJNA234335, and PRJNA287471.
