# Supplementary material for: Identification of key genes affecting porcine fat deposition based on co-expression network analysis of weighted genes
Source: J Anim Sci Biotechnol. 2021 Aug 20;12:100. doi: 10.1186/s40104-021-00616-9 (PMC8379819; doi:10.1186/s40104-021-00616-9)
Supplement: Supplementary file 1 — Additional file 1: Supplementary Table 1. Body weight and backfat thickness between different groups. Supplementary Table 2. KEGG analysis of genes in the four modules related to traits. Supplementary Fig. 1. Analysis of network topology for various soft-thresholding powers. According to the definition of soft threshold, try to choose a large R2 value. According to the suggestion of WGCNA package, choose an R2 value greater than 0.8, that is, the value above the red line in the left figure. The figure on the right shows the average connectivity of the constructed network. The larger the network is, the closer the gene is, and the more conducive it is to screen out hub genes. Supplementary Fig. 2. Venn map of differentially expressed genes (DEGs) of Panel a, b and c respectively shows the situation of the differentially expressed genes in adipose tissue, muscle and liver of Songliao black pigs. Panel e, f and g respectively shows the situation of the differentially expressed genes in adipose tissue, muscle and liver of Landrace. The x-axis represents the multiple of difference, which is denoted by log2FoldChange. The larger the absolute value is, the larger the multiple of difference is. The y-axis represents the significance of the difference, which is denoted by -log10(P-value). The larger the value is, the more significant the difference is. Each panel shows the names of the top 20 genes with the most significant differences. Supplementary Fig. 3. Volcanogram of differentially expressed genes in different tissues of two breeds. Panel a and b respectively shows the overlap of the differentially expressed genes in adipose tissue, muscle and liver of Songliao black pigs and Landrace. Panel b shows the overlap of all the differentially expressed genes of Songliao black pigs and Landrace. Supplementary Fig. 4. GO enrichment analysis and KEGG pathway analysis in different tissues of two breeds. Panel a, b and c respectively shows the enrichment entries of the differenti [file 40104_2021_616_MOESM1_ESM.zip › Supplementary materials4.12.docx]

**Supplementary Figure 1 Analysis of network topology for various soft-thresholding powers**

According to the definition of soft threshold, try to choose a large R^2^ value. According to the suggestion of WGCNA package, choose an R^2^ value greater than 0.8, that is, the value above the red line in the left figure. The figure on the right shows the average connectivity of the constructed network. The larger the network is, the closer the gene is, and the more conducive it is to screen out hub genes.

**Supplementary Figure 2 Venn map of differentially expressed genes (DEGs)**

Venn map of differentially expressed genes (DEGs) of Panel **a**, **b** and **c** respectively shows the situation of the differentially expressed genes in adipose tissue, muscle and liver of Songliao black pigs. Panel **e**, **f** and **g** respectively shows the situation of the differentially expressed genes in adipose tissue, muscle and liver of Landrace. The x-axis represents the multiple of difference, which is denoted by log2FoldChange. The larger the absolute value is, the larger the multiple of difference is. The y-axis represents the significance of the difference, which is denoted by -log10 (*P*-value). The larger the value is, the more significant the difference is. Each panel shows the names of the top 20 genes with the most significant differences

**Supplementary Figure 3 Volcanogram of differentially expressed genes in different tissues of two breeds**

Panel **a** and **b** respectively shows the overlap of the differentially expressed genes in adipose tissue, muscle and liver of Songliao black pigs and Landrace. Panel **b** shows the overlap of all the differentially expressed genes of Songliao black pigs and Landrace

**Supplementary Figure 4 GO enrichment analysis and KEGG pathway analysis in different tissues of two breeds**

Panel **a, b** and **c** respectively shows the enrichment entries of the differentially expressed genes in adipose tissue, liver and muscle of Landrace. Panel **d, e** and **f** shows the enrichment entries of the differentially expressed genes in adipose tissue, liver and muscle of Songliao black pigs. The x-axis represents the significance of the difference, which is denoted by -log2 (P-value). Each bubble represents an enriched function, and the size of the bubble is set with six gradients according to the p-value, from small to large, representing the different significance levels: ns (*P*-value >= 0.05), * (0.01 <= *P*-value < 0.05) , ** (0.001 <= *P*-value < 0.01), *** (0.0001 <= *P*-value < 0.001), **** (1e-10 <= *P*-value < 0.0001), ***** (*P*-value < 1e-10). The color of the bar is the same as the color in the circular network, which represents different clusters. For each cluster, if there are more than 5 terms, top 5 with the highest enrich ratio will be displayed

**Supplementary Table 1 Body weight and backfat thickness between different groups**

| Group | Animal number | Weight, kg | Backfat thickness of measure, mm | Corrected to 100kg of Backfat thickness, mm |
| --- | --- | --- | --- | --- |
| Songliao black pig (High backfat thickness group) | H710 | 116.4 | 24.9 | 21.69 |
|  | H815 | 116.4 | 24.0 | 20.91 |
|  | H906 | 104.8 | 21.7 | 20.80 |
|  | H510 | 92.8 | 17.3 | 18.50 |
|  | H110203 | 116.2 | 28.4 | 24.79 |
|  | H406 | 139.0 | 29.9 | 22.12 |
| Songliao black pig (Low backfat thickness group) | H712 | 108.5 | 9.4 | 8.73 |
|  | H813 | 105.2 | 14.6 | 13.95 |
|  | H909 | 92.5 | 8.8 | 9.44 |
|  | H611 | 85.0 | 5.6 | 6.48 |
|  | H1706 | 96.2 | 7.6 | 7.87 |
|  | H1009 | 76.4 | 6.4 | 8.13 |
| Landrace (High backfat thickness group) | 22511 | 87.4 | 9.7 | 10.94 |
|  | 23712 | 88.4 | 8.8 | 9.83 |
|  | 31210 | 84.3 | 7.6 | 8.85 |
|  | 15805 | 92.7 | 9.4 | 10.06 |
|  | 17608 | 113.6 | 11.4 | 10.15 |
|  | 17109 | 104.5 | 9.7 | 9.32 |
| Landrace (Low backfat thickness group) | 22509 | 78.0 | 3.8 | 4.74 |
|  | 23709 | 81.0 | 3.8 | 4.59 |
|  | 31208 | 93.4 | 3.8 | 4.04 |
|  | 21617 | 101.4 | 2.7 | 2.67 |
|  | 14817 | 95.9 | 2.9 | 3.01 |
|  | 12110 | 90.6 | 2.9 | 3.17 |

The following pairs of individuals are full siblings: H710/H712, H813/H815, H906/H909, 22509/22511, 23709/23711, and 31208/31210.

**Supplementary Table 2** KEGG analysis of genes in the four modules related to traits

| **Module** | **Term** | **Count** | **%** | ***P*-value** |
| --- | --- | --- | --- | --- |
| **black** | ssc03013: RNA transport | 8 | 2.99 | 0.006892542 |
|  | ssc03020: RNA polymerase | 4 | 1.49 | 0.012252829 |
|  | ssc00240: Pyrimidine metabolism | 6 | 2.24 | 0.013290493 |
| **blue** | ssc05166: HTLV-I infection | 55 | 2.62 | 1.0614E-06 |
|  | ssc03013: RNA transport | 36 | 1.71 | 2.47664E-05 |
|  | ssc04810: Regulation of actin cytoskeleton | 42 | 2.00 | 5.72678E-05 |
|  | ssc05205: Proteoglycans in cancer | 41 | 1.95 | 6.05591E-05 |
|  | ssc00562: Inositol phosphate metabolism | 20 | 0.95 | 0.000101204 |
|  | ssc04910: Insulin signaling pathway | 31 | 1.48 | 0.000103429 |
|  | ssc04114: Oocyte meiosis | 27 | 1.29 | 0.00014627 |
|  | ssc05152: Tuberculosis | 37 | 1.76 | 0.000202096 |
|  | ssc04261: Adrenergic signaling in cardiomyocytes | 30 | 1.43 | 0.000218243 |
|  | ssc05169: Epstein-Barr virus infection | 27 | 1.29 | 0.000315377 |
|  | ssc04070: Phosphatidylinositol signaling system | 24 | 1.14 | 0.000324768 |
|  | ssc04932: Non-alcoholic fatty liver disease (NAFLD) | 34 | 1.62 | 0.000390627 |
|  | ssc04919: Thyroid hormone signaling pathway | 26 | 1.24 | 0.00066293 |
|  | ssc05414: Dilated cardiomyopathy | 21 | 1.00 | 0.000722773 |
|  | ssc05410: Hypertrophic cardiomyopathy (HCM) | 20 | 0.95 | 0.000845028 |
|  | ssc05215: Prostate cancer | 21 | 1.00 | 0.000996981 |
|  | ssc05416: Viral myocarditis | 17 | 0.81 | 0.001100177 |
|  | ssc05210: Colorectal cancer | 18 | 0.86 | 0.001144362 |
|  | ssc04722: Neurotrophin signaling pathway | 26 | 1.24 | 0.001286898 |
|  | ssc04914: Progesterone-mediated oocyte maturation | 21 | 1.00 | 0.001355973 |
|  | ssc04150: mTOR signaling pathway | 16 | 0.76 | 0.001522706 |
|  | ssc05142: Chagas disease | 24 | 1.14 | 0.001617888 |
|  | ssc05100: Bacterial invasion of epithelial cells | 19 | 0.90 | 0.00162 |
|  | ssc04068: FoxO signaling pathway | 28 | 1.33 | 0.001642887 |
|  | ssc05010: Alzheimer's disease | 34 | 1.62 | 0.001972383 |
|  | ssc04670: Leukocyte transendothelial migration | 25 | 1.19 | 0.001978715 |
|  | ssc05200: Pathways in cancer | 63 | 3.00 | 0.002008273 |
|  | ssc05221: Acute myeloid leukemia | 15 | 0.71 | 0.002110011 |
|  | ssc05213: Endometrial cancer | 14 | 0.67 | 0.002410135 |
|  | ssc04142: Lysosome | 25 | 1.19 | 0.002520366 |
|  | ssc05145: Toxoplasmosis | 23 | 1.09 | 0.002842285 |
|  | ssc05412: Arrhythmogenic right ventricular cardiomyopathy (ARVC) | 16 | 0.76 | 0.003098256 |
|  | ssc04062: Chemokine signaling pathway | 33 | 1.57 | 0.003141916 |
|  | ssc05212: Pancreatic cancer | 16 | 0.76 | 0.003652828 |
|  | ssc04666: Fc gamma R-mediated phagocytosis | 19 | 0.90 | 0.003948421 |
|  | ssc04015: Rap1 signaling pathway | 37 | 1.76 | 0.004802174 |
|  | ssc04921: Oxytocin signaling pathway | 29 | 1.38 | 0.004851123 |
|  | ssc05012: Parkinson's disease | 29 | 1.38 | 0.004851123 |
|  | ssc04012: ErbB signaling pathway | 19 | 0.90 | 0.005886082 |
|  | ssc05164: Influenza A | 31 | 1.48 | 0.006434684 |
|  | ssc04310: Wnt signaling pathway | 25 | 1.19 | 0.00676474 |
|  | ssc05214: Glioma | 15 | 0.71 | 0.006932532 |
|  | ssc04120: Ubiquitin mediated proteolysis | 26 | 1.24 | 0.007580385 |
|  | ssc05140: Leishmaniasis | 15 | 0.71 | 0.008050862 |
|  | ssc05220: Chronic myeloid leukemia | 16 | 0.76 | 0.008923888 |
|  | ssc04145: Phagosome | 27 | 1.29 | 0.00919246 |
| **brown** | ssc04120: Ubiquitin mediated proteolysis | 29 | 1.86 | 3.58554E-06 |
|  | ssc00190: Oxidative phosphorylation | 26 | 1.67 | 8.82814E-05 |
|  | ssc05012: Parkinson's disease | 27 | 1.74 | 0.000224956 |
|  | ssc04931: Insulin resistance | 20 | 1.29 | 0.001983993 |
|  | ssc03015: mRNA surveillance pathway | 16 | 1.03 | 0.002714105 |
|  | ssc04141: Protein processing in endoplasmic reticulum | 25 | 1.61 | 0.002832158 |
| **turquoise** | ssc01100: Metabolic pathways | 279 | 10.33 | 1.09346E-18 |
|  | ssc04610: Complement and coagulation cascades | 42 | 1.55 | 2.03203E-16 |
|  | ssc01130: Biosynthesis of antibiotics | 66 | 2.44 | 3.60251E-11 |
|  | ssc04976: Bile secretion | 32 | 1.18 | 1.92271E-09 |
|  | ssc04146: Peroxisome | 33 | 1.22 | 1.03726E-07 |
|  | ssc00260: Glycine, serine and threonine metabolism | 18 | 0.67 | 3.07466E-06 |
|  | ssc00220: Arginine biosynthesis | 12 | 0.44 | 2.13128E-05 |
|  | ssc00250: Alanine, aspartate and glutamate metabolism | 16 | 0.59 | 0.000110113 |
|  | ssc01230: Biosynthesis of amino acids | 22 | 0.81 | 0.00014072 |
|  | ssc00140: Steroid hormone biosynthesis | 19 | 0.70 | 0.000384762 |
|  | ssc00380: Tryptophan metabolism | 16 | 0.59 | 0.000438822 |
|  | ssc04640: Hematopoietic cell lineage | 25 | 0.93 | 0.000524168 |
|  | ssc00010: Glycolysis / Gluconeogenesis | 19 | 0.70 | 0.000829152 |
|  | ssc00770: Pantothenate and CoA biosynthesis | 10 | 0.37 | 0.000886784 |
|  | ssc02010: ABC transporters | 16 | 0.59 | 0.001059634 |
|  | ssc01200: Carbon metabolism | 29 | 1.07 | 0.001077858 |
