# Supplementary figures and images for: Identification of key genes affecting porcine fat deposition based on co-expression network analysis of weighted genes
Source: J Anim Sci Biotechnol. 2021 Aug 20;12:100. doi: 10.1186/s40104-021-00616-9 (PMC8379819; doi:10.1186/s40104-021-00616-9)

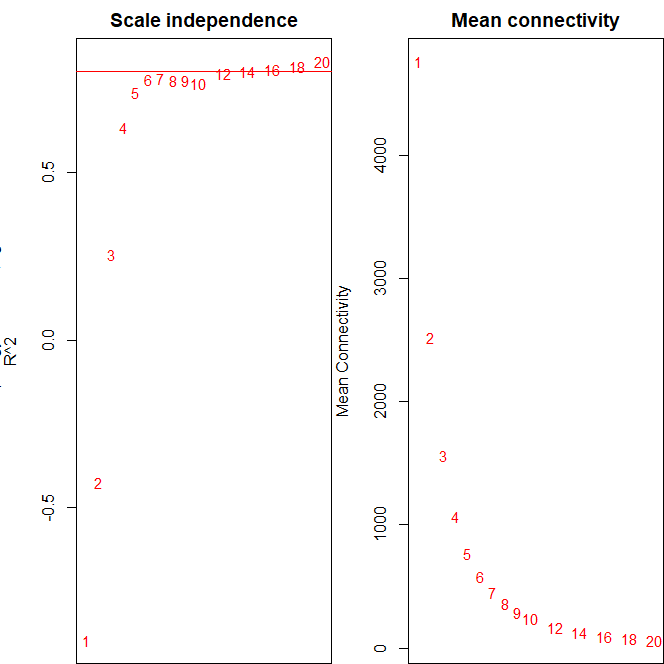

Supplement: Supplementary file 1 — Additional file 1: Supplementary Table 1. Body weight and backfat thickness between different groups. Supplementary Table 2. KEGG analysis of genes in the four modules related to traits. Supplementary Fig. 1. Analysis of network topology for various soft-thresholding powers. According to the definition of soft threshold, try to choose a large R2 value. According to the suggestion of WGCNA package, choose an R2 value greater than 0.8, that is, the value above the red line in the left figure. The figure on the right shows the average connectivity of the constructed network. The larger the network is, the closer the gene is, and the more conducive it is to screen out hub genes. Supplementary Fig. 2. Venn map of differentially expressed genes (DEGs) of Panel a, b and c respectively shows the situation of the differentially expressed genes in adipose tissue, muscle and liver of Songliao black pigs. Panel e, f and g respectively shows the situation of the differentially expressed genes in adipose tissue, muscle and liver of Landrace. The x-axis represents the multiple of difference, which is denoted by log2FoldChange. The larger the absolute value is, the larger the multiple of difference is. The y-axis represents the significance of the difference, which is denoted by -log10(P-value). The larger the value is, the more significant the difference is. Each panel shows the names of the top 20 genes with the most significant differences. Supplementary Fig. 3. Volcanogram of differentially expressed genes in different tissues of two breeds. Panel a and b respectively shows the overlap of the differentially expressed genes in adipose tissue, muscle and liver of Songliao black pigs and Landrace. Panel b shows the overlap of all the differentially expressed genes of Songliao black pigs and Landrace. Supplementary Fig. 4. GO enrichment analysis and KEGG pathway analysis in different tissues of two breeds. Panel a, b and c respectively shows the enrichment entries of the differenti [file 40104_2021_616_MOESM1_ESM.zip › SFig.1.tif]

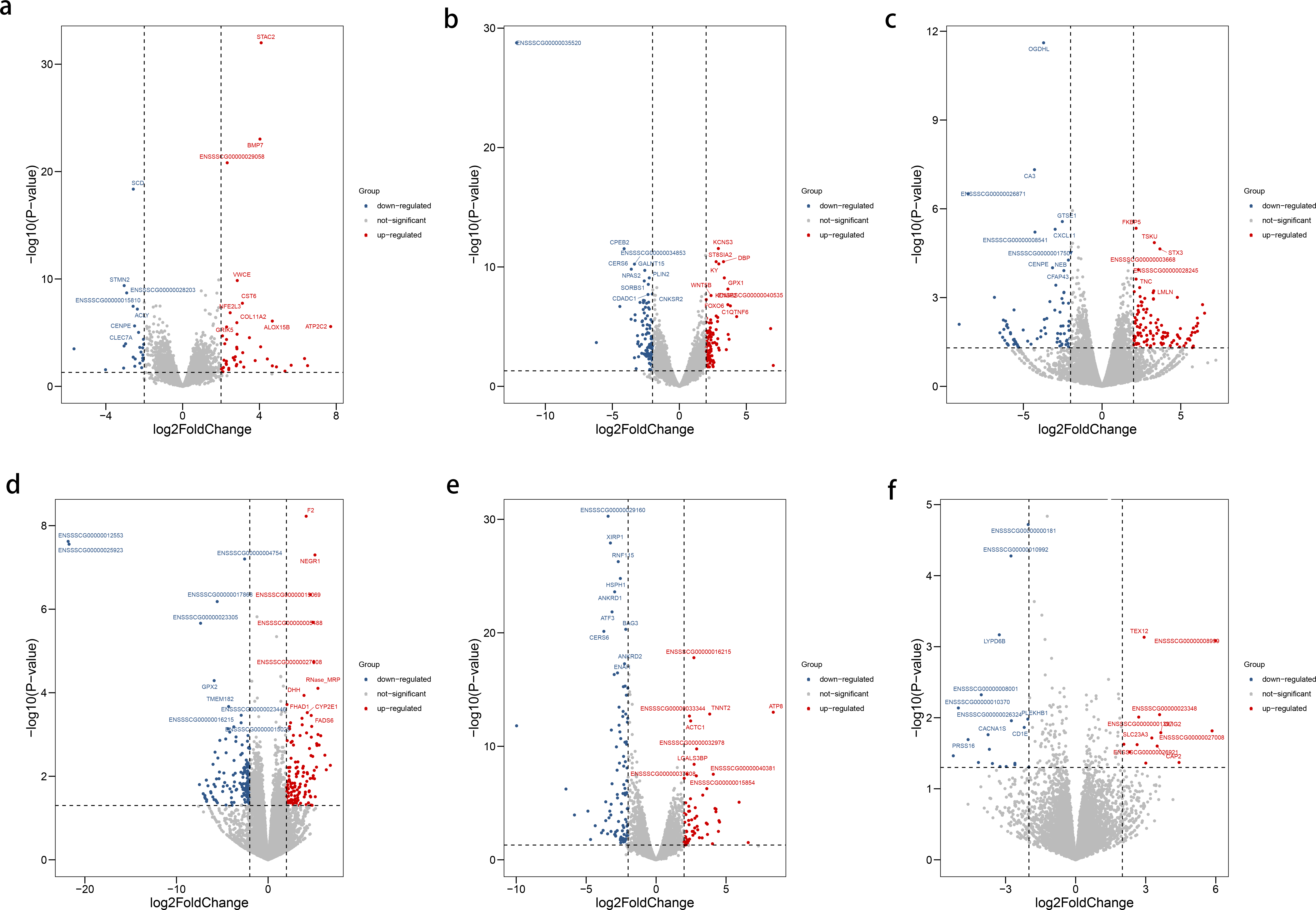

Supplement: Supplementary file 1 — Additional file 1: Supplementary Table 1. Body weight and backfat thickness between different groups. Supplementary Table 2. KEGG analysis of genes in the four modules related to traits. Supplementary Fig. 1. Analysis of network topology for various soft-thresholding powers. According to the definition of soft threshold, try to choose a large R2 value. According to the suggestion of WGCNA package, choose an R2 value greater than 0.8, that is, the value above the red line in the left figure. The figure on the right shows the average connectivity of the constructed network. The larger the network is, the closer the gene is, and the more conducive it is to screen out hub genes. Supplementary Fig. 2. Venn map of differentially expressed genes (DEGs) of Panel a, b and c respectively shows the situation of the differentially expressed genes in adipose tissue, muscle and liver of Songliao black pigs. Panel e, f and g respectively shows the situation of the differentially expressed genes in adipose tissue, muscle and liver of Landrace. The x-axis represents the multiple of difference, which is denoted by log2FoldChange. The larger the absolute value is, the larger the multiple of difference is. The y-axis represents the significance of the difference, which is denoted by -log10(P-value). The larger the value is, the more significant the difference is. Each panel shows the names of the top 20 genes with the most significant differences. Supplementary Fig. 3. Volcanogram of differentially expressed genes in different tissues of two breeds. Panel a and b respectively shows the overlap of the differentially expressed genes in adipose tissue, muscle and liver of Songliao black pigs and Landrace. Panel b shows the overlap of all the differentially expressed genes of Songliao black pigs and Landrace. Supplementary Fig. 4. GO enrichment analysis and KEGG pathway analysis in different tissues of two breeds. Panel a, b and c respectively shows the enrichment entries of the differenti [file 40104_2021_616_MOESM1_ESM.zip › SFig.2.tif]

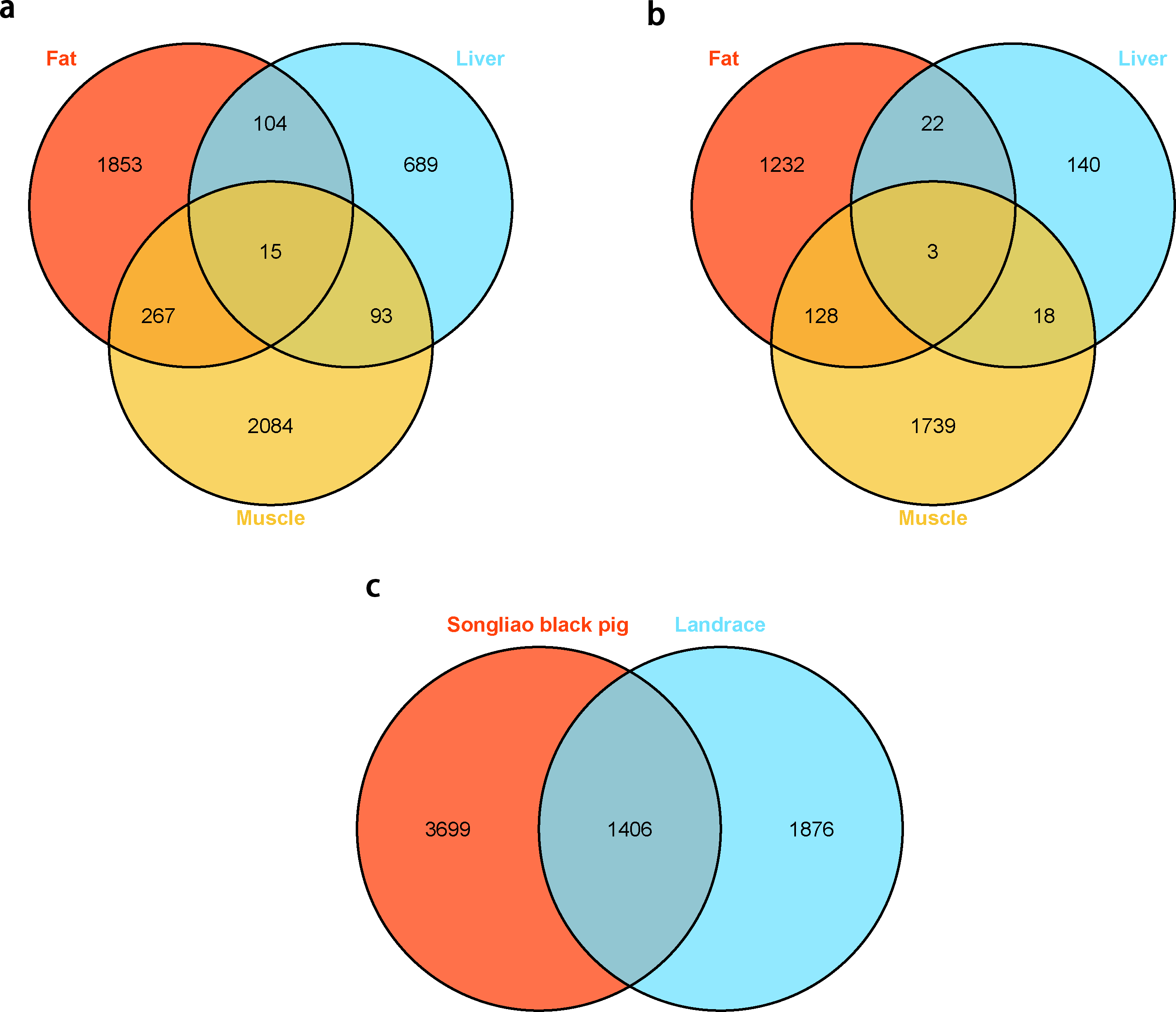

Supplement: Supplementary file 1 — Additional file 1: Supplementary Table 1. Body weight and backfat thickness between different groups. Supplementary Table 2. KEGG analysis of genes in the four modules related to traits. Supplementary Fig. 1. Analysis of network topology for various soft-thresholding powers. According to the definition of soft threshold, try to choose a large R2 value. According to the suggestion of WGCNA package, choose an R2 value greater than 0.8, that is, the value above the red line in the left figure. The figure on the right shows the average connectivity of the constructed network. The larger the network is, the closer the gene is, and the more conducive it is to screen out hub genes. Supplementary Fig. 2. Venn map of differentially expressed genes (DEGs) of Panel a, b and c respectively shows the situation of the differentially expressed genes in adipose tissue, muscle and liver of Songliao black pigs. Panel e, f and g respectively shows the situation of the differentially expressed genes in adipose tissue, muscle and liver of Landrace. The x-axis represents the multiple of difference, which is denoted by log2FoldChange. The larger the absolute value is, the larger the multiple of difference is. The y-axis represents the significance of the difference, which is denoted by -log10(P-value). The larger the value is, the more significant the difference is. Each panel shows the names of the top 20 genes with the most significant differences. Supplementary Fig. 3. Volcanogram of differentially expressed genes in different tissues of two breeds. Panel a and b respectively shows the overlap of the differentially expressed genes in adipose tissue, muscle and liver of Songliao black pigs and Landrace. Panel b shows the overlap of all the differentially expressed genes of Songliao black pigs and Landrace. Supplementary Fig. 4. GO enrichment analysis and KEGG pathway analysis in different tissues of two breeds. Panel a, b and c respectively shows the enrichment entries of the differenti [file 40104_2021_616_MOESM1_ESM.zip › SFig.3.tif]
